# Platelet Transforming Growth Factor-β1 Induces Liver Sinusoidal Endothelial Cells to Secrete Interleukin-6

**DOI:** 10.3390/cells9051311

**Published:** 2020-05-25

**Authors:** Alexandre Balaphas, Jeremy Meyer, Remo Perozzo, Magali Zeisser-Labouebe, Sarah Berndt, Antoine Turzi, Pierre Fontana, Leonardo Scapozza, Carmen Gonelle-Gispert, Leo H. Bühler

**Affiliations:** 1 Division of Digestive Surgery, University Hospitals of Geneva, Rue Gabrielle-Perret-Gentil 4, 1205 Geneva, Switzerland; jeremy.meyer@hcuge.ch; 2 Unit of Surgical Research, University of Geneva, Rue Michel-Servet 1, 1206 Geneva, Switzerland; 3 Pharmaceutical Biochemistry Group, School of Pharmaceutical Sciences, University of Geneva, 1211 Geneva, Switzerland; magali.zeisser@unige.ch (M.Z.-L.); leonardo.scapozza@unige.ch (L.S.); 4 Institute of Pharmaceutical Sciences of Western Switzerland, University of Geneva, 1211 Geneva, Switzerland; 5 Regen Lab SA, En Budron b2, 1052 Le Mont-sur-Lausanne, Switzerland; Sarah.Berndt@unige.ch (S.B.); aturzi@regenlab.com (A.T.); 6 Division of Angiology and Haemostasis, University Hospitals of Geneva, Rue Gabrielle-Perret-Gentil 4, 1205 Geneva, Switzerland; pierre.fontana@hcuge.ch; 7 Geneva Platelet Group, University of Geneva, Rue Michel-Servet 1, 1206 Genève, Switzerland; 8 Faculty of Science and Medicine, Section of Medicine, University of Fribourg, Route Albert-Gockel 1, 1700 Fribourg, Switzerland; carmen.gonelle@unifr.ch (C.G.-G.); leo.buhler@unifr.ch (L.H.B.)

**Keywords:** livers sinusoidal cells, hepatocytes, liver regeneration, Interleukin-6, transforming growth factor β, von Willebrand factor

## Abstract

The roles and interactions of platelets and liver sinusoidal endothelial cells in liver regeneration are unclear, and the trigger that initiates hepatocyte proliferation is unknown. We aimed to identify the key factors released by activated platelets that induce liver sinusoidal endothelial cells to produce interleukin-6 (IL-6), a cytokine implicated in the early phase of liver regeneration. We characterized the releasate of activated platelets inducing the in vitro production of IL-6 by mouse liver sinusoidal endothelial cells and observed that the stimulating factor was a thermolabile protein. Following gel filtration, a single fraction of activated platelet releasate induced a maximal IL-6 secretion by liver sinusoidal endothelial cells (90.2 ± 13.9 versus control with buffer, 9.0 ± 0.8 pg/mL, *p* < 0.05). Mass spectroscopy analysis of this fraction, followed by in silico processing, resulted in a reduced list of 18 candidates. Several proteins from the list were tested, and only recombinant transforming growth factor β1 (TGF-β1) resulted in an increased IL-6 production up to 242.7 ± 30.5 pg/mL, which was comparable to non-fractionated platelet releasate effect. Using neutralizing anti-TGF-β1 antibody or a TGF-β1 receptor inhibitor, IL-6 production by liver sinusoidal endothelial cells was dramatically reduced. These results support a role of platelet TGF-β1 β1 in the priming phase of liver regeneration.

## 1. Introduction

Despite their lack of nuclei, platelets are implicated in numerous functions, including tissue regenerative processes following their activation, which induces their degranulation and release of platelets microparticles (PMP) [1,2,3]. Platelets have been proposed as actors of liver regeneration, although the precise mechanisms have not been unveiled. After partial hepatectomy, platelets accumulate in sinusoids or are translocated into the space of Disse, which separates the liver sinusoidal endothelial cells (LSEC) lining from hepatocytes [2]. Platelets may act through the release of granule-derived proliferative factors or by mechanisms such as platelet internalization by hepatocytes [4] or LSEC [5,6].

Platelets act at several levels in liver regeneration. Direct effects on hepatocyte proliferation were demonstrated through the release of dense granule-derived serotonin [7,8] and α-granules-derived hepatocyte growth factor, vascular endothelial growth factor and insulin-like growth factor-1 [9]. Platelets have also been proposed to interact with non-parenchymal cells, such as LSEC, to drive liver regeneration [1,10,11,12]. We and others demonstrated that activated platelet or activated platelets releasate (APR) induced IL-6 secretion from mouse or human LSEC [13,14,15].

IL-6 is a key cytokine at the center of inflammatory processes and connects acute liver inflammation to liver regeneration [2,16]. It is also crucial to the initiation of the liver regeneration process, as demonstrated by the absence of liver regeneration in IL-6 knock-out [17], and seems to regulate liver regeneration at two levels. First, IL-6 directly acts on hepatocytes through its cognate surface receptors or through free receptors that bind IL-6 and further drives signaling and signal transduction by the activation of the JAK/STAT, ERK and PI3K pathways [18,19,20]. These steps are critical for the hepatocyte cell cycle progression beyond phase G1 [17,18]. Additionally, IL-6 may promote the production of hepatocyte growth factor (HGF) by Kupffer cells (KC) [21] and hepatic stellate cells (HSC) [22], which is critical in the regulation of liver growth and regeneration [23].

Classically, IL-6 is mainly produced by KC after activation by various stimuli [17]. In this study, based on earlier observations that IL-6 is secreted by LSEC upon interactions with platelets, we analyze platelet releasates to unveil the responsible factor(s) of IL-6 secretion by LSEC [13].

## 2. Materials and Methods

### 2.1. Animals

We used 8- to 16-weeks-old male C57BL/6 mice that were obtained from Janvier Labs (Le Genest-Saint-Isle, France). The presented procedures were performed in accordance with protocols approved by the animal ethics committee of the Geneva Veterinarian Office (Geneva, Switzerland). Mice were maintained under standard conditions at the animal facility of the University of Geneva until utilization. Blood from mice deficient in von Willebrand factor (vWF) (double knockout—ko) on a C57BL/6 genetic background was generously provided by Dr. Cecile Denis at the centre hospitalier universitaire Kremlin-Bicêtre, Paris.

### 2.2. Proteins

Purified bovine thrombin, recombinant thrombospondin 1 (TSP1) and SB 431542 hydrate were obtained from Sigma-Aldrich (Buchs, Switzerland). Highly purified full-length free human vWF (#V2651) and thrombin receptor activator peptide 6 (TRAP-6) were obtained from United States Biological (Salem, MA, USA). Recombinant human TGF-β1 and Interleukin-1β1 were obtained from PreproTech (Rocky Hill, NJ, USA). Prostaglandin I2 was purchased from Cayman chemical (Ann Arbor, MI, USA).

### 2.3. Materials

A QuadraMACS separator, a MACS MultiStand platform, CD11b microbeads for mice and LS columns were acquired from Miltenyi Biotec (Bergisch Gladbach, Germany). Amicon ultra 0.5- and 4-mL centrifugal filters were purchased from Merck Millipore (Billerica, MA, USA). A Protein determination Bradford kit was purchased from BioRad (Hercules, CA, USA). ELISA kits for mouse IL-6, human IL-6 and human vWF were obtained from RayBiotech (Norcross, GA, USA). ELISA kits for human total transforming growth factor β1 (TGF-β1) were obtained from Biolegend (San Diego, CA, USA). Regen BCT tubes were generously supplied by Regen Lab (Le Mont-sur-Lausanne, Switzerland). Falcon tubes and 70 μm cell strainers were obtained from Corning (Corning, NY, USA). Centrifuges included a Rotixa 50 RS (Hettich Zentrifugen, Tuttlingen, Germany) and an Eppendorf Centrifuge 5415 R (Sigma-Aldrich, Buchs, Switzerland). Flow cytometry sample acquisition was performed with an Accuri C6 (Becton Dickinson, Franklin Lakes, NJ, USA). A NuPAGE kit for gel electrophoresis of protein and silver stain was purchased from Thermo Fisher Scientific (Waltham, MA, USA).

### 2.4. Reagents

Solutions for liver isolation were always freshly prepared and filtered at 0.22 μm, as previously described by Meyer et al. [24]. Wash solution: HEPES buffer, penicillin-streptomycin, EGTA from Life Technologies (Grand Island, NY, USA), glucose 40% from Grosse Apotheke Dr. G. Bichsel AG (Interlaken, Switzerland) and high-molecular-weight heparin from Drossapharm (Arlesheim, Switzerland) in Hank’s balanced salt solution without calcium from Life Technologies (Grand Island, NE, USA). Digestion solution: type 4 collagenase 270 units/mg from Worthington Biochemical Corporation (Lakewood, USA), DNase 2000 units/mg from Roche Diagnostics (Risch, Switzerland), in William’s E with Glutamax from Life Technologies (Grand Island, NY, USA). STOP solution: fetal bovine serum (FBS), penicillin–streptomycin from Life Technologies (Grand Island, NY, USA) in William’s E with Glutamax medium. MACS buffer: EDTA from Promega, (Madison, WI, USA) with FBS in Calcium-free Dulbecco’s phosphate-buffered saline (DPBS) from Life Technologies (Grand Island, NY, USA). Tyrode buffer was made on the day of each experiment using cell culture grade NaCl, Hepes Buffer, Glucose, KCl, NaHCO_3_, Na_2_HPO4 and MgCl_2_ from Sigma-Aldrich (Buchs, Switzerland). Tricine solution was prepared from tricine and NaCl obtained from Sigma-Aldrich (Buchs, Switzerland). OptiPrep density gradient separation solution was purchased from Axis-Shield (Oslo, Norway). Endothelial cell Medium was acquired from CellBiologics (Chicago, MI, USA). Calcium buffer was prepared with HEPES, NaCl and CaCl_2_ obtained from Sigma-Aldrich (Buchs, Switzerland).

### 2.5. Antibodies

Alex Fluor 488-conjugated anti-mouse stabilin-2 was obtained from MBL International (Woburn, MA, USA). Polyclonal anti-mouse IBA-1 was obtained from Dako/Agilent Technologies (Santa Clara, CA, USA) Allophycocyanin-conjugated anti-mouse F4/80, fluorescein-conjugated annexin V and allophycocyanin-conjugated anti-human CD41 or G1κ isotype were purchased from Biolegend (San Diego, CA, USA). Purified anti-human vWF was obtained from Dako/Agilent Technologies (Santa Clara, CA, USA). Purified anti-human TGF-β1 was acquired from GenTex (Irvine, CA, USA) and Biolegend (San Diego, CA, USA). Anti-mouse IL-6 was obtained from RayBiotech (Norcross, GA, USA). Alexa Fluor 488 or Alexa fluor 555-conjugated anti-rat/rabbit antibodies were obtained from Invitrogen (Carlsbad, CA, USA). AF488 with Hoechst was obtained from Thermo Fisher Scientific (Waltham, MA, USA).

### 2.6. Isolation and Culture of Liver Sinusoidal Cells, Kupffer Cells or HUVEC

IL-6 secretion assays were repeated as independent experiments at least twice, if not otherwise specified. LSEC were isolated and cultured according to the protocol previously reported [24]. KC and HSC were retrieved using an adaptation of LSEC isolation protocol. Briefly, after cannulation of the supra-hepatic portion of the inferior vena through the right atrium, the mouse liver was rinsed with wash solution and digested in situ with heated digestion solution. After digestion, the liver was further disrupted mechanically, and the enzymatic reactions were blocked with the STOP solution. Digested tissue was filtered to remove cell clusters. Hepatocytes were removed by centrifugation, and LSEC were extracted from non-parenchymal cells with density separation solution made from Optiprep and Tricine solution. Retrieved cells were suspended in MACS buffer and incubated with CD11b beads, and contaminant CD11b+ macrophages were removed using magnetic-activated cell sorting (MACS). LSEC yield was assessed with Neubauer chamber and purity by flow cytometry for Stabilin-2 and F4/80 expression. Only preparations with less than 5% contaminant macrophages were used for experiments. LSEC were plated for culture with endothelial cell medium at a density of 100,000 LSEC/well in a 96 well plate. LSEC were rinsed at 12 h of adherence, and at 24 h of culture, they were incubated with 125 µL of William’s E 1% FBS and 25 µL of APR or control for a further 24 h. For experiments with recombinant proteins, a mixture of William’s E and the protein of interest was prepared. For neutralizing experiments, antibodies were added to APR or control buffer one hour prior to utilization of the LSEC. The serine–threonine inhibitor SB431543, acting on the LSEC, was directly incubated with LSEC before adding APR or control.

For KC preparation, the same isolation procedure was performed as described, and CD11b+ macrophages retained in the MACS column were flushed with 4 mL of MACS buffer. The suspension was then centrifuged, and KC pellets were suspended in Roswell Park Memorial Institute medium with 10% FBS. KC yield was assessed with Neubauer chamber, and cells were plated as microdrops of 25,000 cells for immunofluorescence staining.

Human umbilical cord endothelial cells (HUVEC), donated by V. Serre-Benier, were cultured until fifth passage in Medium 199 from Thermo Fisher Scientific (Waltham, MA, USA) with 20% FBS and endothelial cell growth supplement purchased from Sigma-Aldrich (Buchs, Switzerland). HUVEC were plated in 96 wells with a density of 10,000 cells/well and incubated similarly to the LSEC, either with 150 μL William’s E, 1% FCS and 25 μL APR or 25 µL control medium for 24 h.

### 2.7. Preparation of Mouse Activated Platelet Releasate

Mouse platelets and APR were prepared as previously described [13]. Mouse APR was prepared from the blood of 3 to 4 mice retrieved after cardiac puncture. After 120 µL sodium citrate–dextrose was added to the blood of each mouse, samples were pooled and centrifuged at 100× *g* for 15 min three times to obtain platelet-rich plasma (PRP). Platelets were separated from plasma by centrifugation at 600× *g* for 15 min and then suspended in warm tyrode buffer. Their yield was evaluated with Neubauer chamber.

### 2.8. Preparation of Platelet Microparticles

PMP were prepared by activation of human platelets with either TRAP-6 with a final concentration of 20 µM or by freeze-thawing (−80 °C freezing for at least 24 h, followed by rapid thawing in a bain-marie at 37 °C). All centrifugations were run at room temperature. After activation, the platelet solution was centrifuged at 5000× *g* for 5 min followed by 11,000× *g* for 1 min. After this step, supernatant was retrieved and further centrifuged 2500× *g* for 15 min. Supernatant was again retrieved and centrifuged at 15,000× *g* for 90 min to pull down PMP. Pellets were suspended in 500 μL PBS and washed once with a new round at 15,000× *g* for 90 min. After this last step, pellets were suspended in 100 μL of PBS, and tubes were pooled. For flow cytometry, platelets, platelet supernatants or PMP preparations were diluted twice and incubated with allophycocyanin-conjugated anti-human CD41 or G1k isotype diluted 1/10 for 15 min. The preparation was further diluted 1/5 with Calcium buffer, and Annexin V diluted 1/100 was added.

### 2.9. Platelet Releasate Preparation and Fast Protein Liquid Chromatography

For human APR preparation, 10–20 mL of blood was retrieved from four different healthy volunteers after informed consent using the Regen BCT tubes containing citrate sodium. Tubes were gently inverted twice and centrifuged at 1500× *g* for 5 min at room temperature. The retrieved tubes were gently inverted 20 times to homogenize the separated PRP. Afterward, platelets were left in suspension for one hour at room temperature before collection of the PRP. We collected between 4 and 6 mL of PRP from each tube. After adding 2.5 µL/mL PGI2, platelets were pulled down with centrifugation at 2200× *g* for 14 min. Platelets were then suspended in tyrode buffer, and their number was estimated with Neubauer chamber. Mouse or human platelet suspension was adjusted to a concentration of either 320,000 or 640,000 platelets/µL. One hour after PGI_2_ had been added to the platelets, activation was performed with thrombin 1 U/mL for 30 min. The suspension was divided in aliquots of 500 μL and was further centrifuged at 600× *g* or 2200× *g* for 45 min, for mouse and human platelets. After this step, APR was collected. For the PMP depletion experiment, APR was centrifuged at 16,000× *g* for 60 min.

Before each experiment, the whole Äkta pure chromatography system (GE Helthcare, Chicago, IL, USA), including the tubing, was washed with a successive perfusion of NaOH 1M and IGEPAL CA-630 0.1% from Sigma-Aldrich (Buchs, Switzerland). Each human APR sample was first concentrated to a volume of 500 µL with Amicon 10 kDa 4 mL tube and centrifuged at 18,000× *g* for 2 min to remove residual aggregates or cells. Tyrode buffer was used as running buffer to equilibrate the Superdex 75 Increase column (GE Helthcare, Chicago, IL, USA). The sample was then injected using a 1 mL loop. Elution was performed over 1.5× column volume, and the first 25 fractions of 1 mL were collected.

### 2.10. Western Blot

Proteins were extracted from 64,000 HUVEC, 300,000 LSEC or 300,000 KC. Proteins were separated by electrophoresis in a sodium dodecyl sulfate (Invitrogen, Taastrup, Denmark) polyacrylamide gel. Different gel densities were used according to the size of the investigated protein. The samples were subsequently transferred onto polyvinylamide fluoride membranes (Hybond-P, GE Healthcare, Little Chalfont, UK) and blocked with blocking buffer (Tris-HCl (pH 7.6)) containing 150 mmol/L NaCl, 0.1% Tween-20 and 5% non-fat dry milk). Primary antibodies were incubated overnight at 4 °C in blocking buffer. Then, the membranes were rinsed with TBS-Tween and incubated with a goat anti-mouse or anti-rabbit secondary antibody (Hercules, CA, USA) conjugated to horseradish peroxidase and diluted in blocking buffer. An enhanced chemiluminescence detecting kit (Amersham Pharmacia Biotech, Piscataway, NJ, USA) was used to read the membranes. Protein load was controlled after probing the membranes with a rabbit polyclonal antibody directed against β-actin.

### 2.11. Immunofluorescence

For immunofluorescence staining, cells were plated on a glass coverslip and incubated with lipopolysaccharide 0.5 mg/mL (Sigma-Aldrich Buchs, Switzerland) or control media. Coverslips were fixed with paraformaldehyde from Sigma-Aldrich (Buchs, Switzerland) and further permeabilized with 0.2% Triton-X-100 from Sigma-Aldrich (Buchs, Switzerland) for 15 min. After blocking of nonspecific binding sites with PBS/3% BSA/10% goat serum for 1 h, coverslips were incubated with primary antibodies overnight. The following day, cells were washed and incubated with secondary antibody for 2 h. At the end of incubation, nuclei were stained with Hoechst for 10 min. Images were acquired with an LSM800 Airyscan (Zeiss, Oberkochen, Germany) using an Apochromat 63×/1.40 Oil M27 objective or a Leica DFC320 camera microscope (Wetzlar, Germany).

### 2.12. Data Analysis

Confocal images were analyzed with a Zen 2.3 SP1 (Zeiss, Oberkochen, Germany). Flow cytometry data were analyzed with FlowJo 10 (FlowJO LLC, Ashland, OR, USA). Mass spectroscopy (MS) data were analyzed with Scaffold4, (Proteome Software, Portland, OR, USA). In silico determination of protein identities was performed with the online database and tools of UniProt (European Bioinfirmatics Institue, Swiss Institue of Bioinformatics and Protein Information Ressource). Protein polyacrylamide gel electrophoresis pictures were acquired and processed using FusionCapt version 17.03 (Witec, Sursee, Switzerland).

### 2.13. Statistics

Data graphical representation, descriptive statistics and statistical tests were performed with GraphPad Prism version 8 (GraphPad Software Inc., La Jolla, CA, USA). Means were expressed with their standard deviation (mean ± SD). We used t-tests to compare two conditions in all experiments, including titration assays. Indeed, heteroscedasticity and limited number of replicas per independent experiment precluded the utilization, respectively, of ANOVA and Kruskal-Wallis tests for titration experiments. An alpha level of 0.05 was set for statistical significance.

### 2.14. Study Approval

The study approval was granted by the animal ethics committee of the Geneva Veterinarian Office (Geneva, Switzerland). All procedures performed in this study involving human participants were in accordance with the ethical standards of the 1964 Helsinki Declaration and its later amendments or comparable ethical standards.

## 3. Results

### 3.1. Activated Platelet Releasate Induces Interleukin-6 Expression and Secretion in Liver Sinusoidal Endothelial Cells

**Figure 1:**
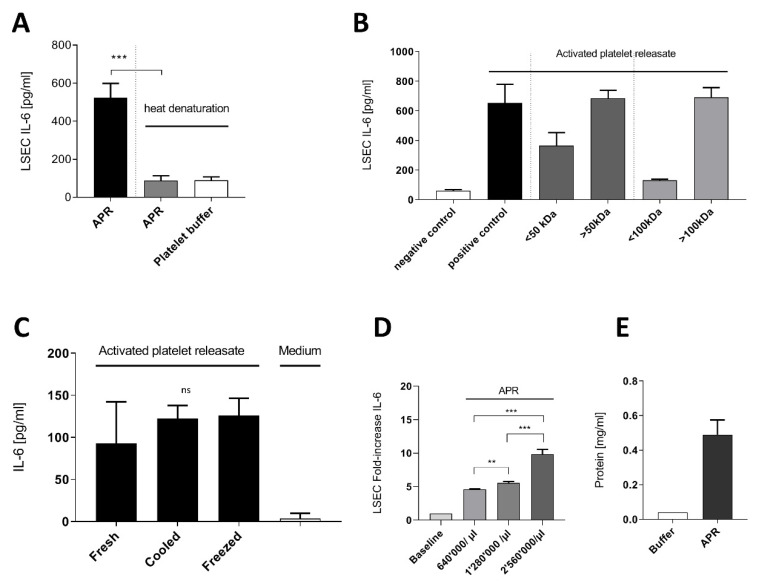
Characterization of activated platelet releasate soluble factor. (**A**): Mouse activated platelets releasate (APR) demonstrated lability to heat denaturation (95 °C, 15 min). (**B**): Using Amicon centrifugation filter of 50 kDa and 100 kDa cutoff, the supernatants containing proteins ˃ 50 kDa induced IL-6 secretion (**C**): Freshly prepared APR (Fresh) and after storage at 4 °C (Cooled) or at −20 °C (Frozen) still induced IL-6 secretion in LSEC. (**D**): Increasing platelet concentration in the preparation resulted in a significant fold increase of IL-6 production by LSEC. Baseline corresponds to basal IL-6 production by LSEC (**E**): Bradford assay estimating the total protein concentration of two mouse APR preparation of 640,000 platelet/µL. Buffer corresponds to platelet buffer. Presented data are representative of 2 or 3 independent experiments. *** *p*-value between 0.0001 and 0.001, ** *p*-value between 0.001 and 0.01 (*t*-test), ns: non-significant.

Through heat denaturation and protein concentration (50k Da to 100k Da cut-off Amicon filters) experiments with mouse thrombin APR, we confirmed that the APR active soluble factor is thermolabile and of high molecular weight (Figure 1A,B), suggesting a peptidic nature. Further, the APR soluble factor preserved its activity on LSEC after 4 °C storing for 24 h or freezing (Figure 1C). As described in a previous work, incubation of 640,000 mouse platelet/µL for 100,000 LSEC was the optimal concentration to observe a significant increased IL-6 secretion by LSEC [13]. Thus, we prepared mouse APR from different platelet concentrations to investigate whether LSEC were able to increase IL-6 production. Indeed, we demonstrated that LSEC still increased their IL-6 production up to a mean fold increase of 9.8 ± 0.73 with 2,560,000 platelets/µL (Figure 1D). Moreover, mean APR protein concentration was estimated to be 0.49 mg/mL ± 0.085 in 640,000 mouse platelet/µL (Figure 1E).

Further, human APR induced a threefold production of IL-6 by LSEC compared to mouse APR when prepared with the same platelet concentrations (Figure S1A). Moreover, we observed a statistically significant increase of IL-6 secretion by HUVEC following stimulation with either mouse or human APR compared to control (Figure S1B).

**Figure 2:**
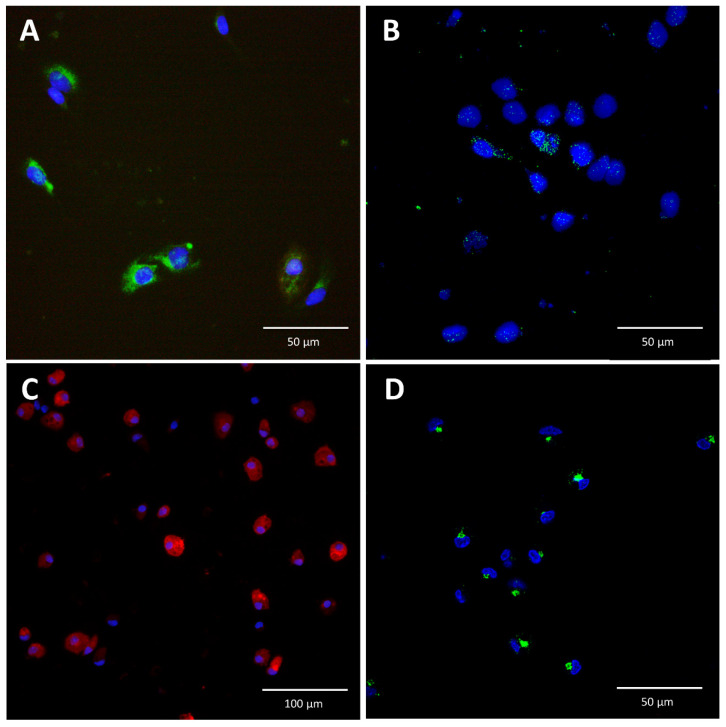
Immunofluorescence staining for interleukin-6 in liver sinusoidal endothelial cells and Kupffer cells. (**A**): Isolated liver sinusoidal endothelial cells (LSEC) were stained for stabilin-2 (green), and their nuclei were stained with Hoechst (blue). (**B**): In the absence of stimulation, IL-6 was not detected in LSEC (not shown). In contrast, after incubation of LSEC with APR, IL-6 (green) was detected in vesicles, with a homogeneous distribution in the cytoplasm. (**C**): Isolated Kupffer cells (KC) were stained for IBA-1 (red) and their nuclei were stained with Hoechst (blue). (**D**): KC were stimulated with lipopolysaccharide (0.5 mg/mL) and exhibited a different cytoplasmic distribution of IL-6 than LSEC. In LSEC, IL-6 was more homogeneously distributed in the cytoplasm (**B**) around the nuclei, whereas in KC, IL-6 appeared in clustered vesicles (**D**).

Using immunofluorescence staining, we demonstrated that IL-6 is expressed in LSEC and is detectable after stimulation of LSEC with APR (Figure 2). We found that the cellular pattern of IL-6 distribution was different in LSEC compared to KC. In KC, IL-6 appeared in clustered vesicles (Figure 2D), whereas in LSEC, IL-6 was more homogeneously distributed in the cytoplasm around the nuclei (Figure 2B).

### 3.2. Platelet-Derived Extracellular Vesicles Are Not Implicated in Interleukin-6 Secretion by Liver Sinusoidal Endothelial Cells

Extracellular vesicles (EV) can vehicle molecules, including proteins. To analyze whether supernatants of mouse APR, depleted from platelet PMP, remain active and stimulate LSEC IL-6 secretion, we selectively removed PMP by differential centrifugation. After centrifugation, mouse APR supernatant containing proteins and exosomes still increased IL-6 secretion by LSEC and was similar to the control conditions (Figure S2A). Moreover, using PMP prepared from TRAP-6 human APR or derived from human platelet submitted to freeze-thawing, an increase of IL-6 production by LSEC was not observed (Figure S2B,C). PMP preparations were qualitatively assessed with flow cytometry. PMP were defined by events that did not have platelet morphology on the side and forward scatter and were double-positive for allophycocyanin-conjugated human anti-CD41 and fluorescein-conjugated annexin V (Figure S2D,E).

### 3.3. Identification by Fast Protein Liquid Chromatography of a Single Fraction Inducing Interleukin-6 Secretion in Liver Sinusoidal Endothelial Cells

**Figure 3:**
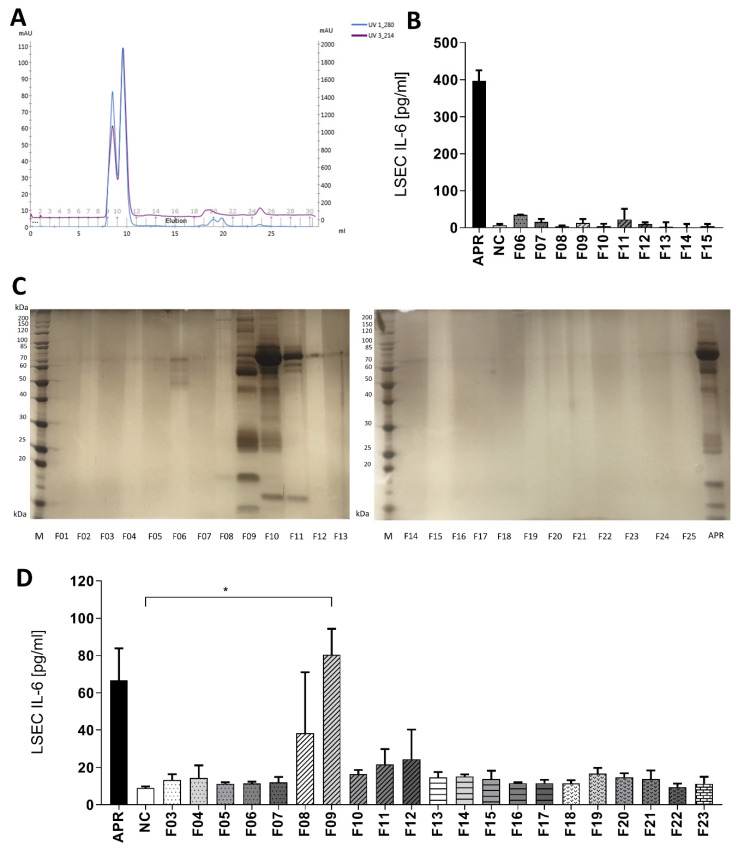
Gel filtration on a Superdex 75 increase column. (**A**): UV protein absorbance at 280 or 214 nm. All proteins of the sample were eluted from fraction 09 to 12. (**B**): Control experiment: eluted fractions of applied tyrode buffer and purified bovine thrombin were used for inducing IL-6 secretion by LSEC. Thrombin in tyrode buffer fractions did not activate LSEC. NC: negative control, tyrode buffer. (**C**): Silver-stained 12% polyacrylamide gel after SDS-PAGE showing proteins in APR fractions obtained after gel filtration. Proteins were mainly found in fractions 09 to 12. M: calibration, APR: non-fractionated APR. (**D**): Selected fractions were added to LSEC and IL-6 secretion assessed after 24 h of culture. Solely fraction 09 (F09) induced a statistically different IL-6 response compared to tyrode buffer, the negative control (NC), * *p*-value between 0.01 and 0.05 (*t*-test). APR prepared from 640,000 platelets/µL. *n* = 2. If not otherwise specified, presented data are representative of 2 or 3 independent experiments.

To identify the platelet soluble factors (for simplification, we will further refer to a single soluble factor in the text), we ran a fast protein liquid chromatography (FPLC) using a size exclusion column of 75 KDa. Maximal absorbance at 280 nm and 214 nm was observed in fractions 09 to 12, indicating that the majority of the APR proteins were distributed in between these fractions (Figure 3A). After collection of the 25 × 1 mL fractions and loading on a 12% SDS-page gel stained with silver, proteins were mainly found in fractions 09 to 12 (Figure 3B). To exclude the presence of contaminating proteins in the platelet buffer, we repeated this experiment with a solution of thrombin diluted in tyrode buffer, and no protein bands were detected (data not shown).

Further, the obtained human APR fractions or tyrode buffer/thrombin fractions were added on LSEC cultures to analyze their biological activity. We observed that only fractions 08 and 09 induced IL-6 secretion by LSEC. However, fraction 09 triggered the highest response in two independent experiments and induced a statistically significant increase of IL-6 production by LSEC compared to negative control (90.2 ± 13.9 versus 9.0 ± 0.8 pg/mL, *p* < 0.05) (Figure 3D). Finally, tyrode buffer/thrombin fractions did not stimulate IL-6 production by LSEC (Figure 3B).

Mass spectroscopy analysis of fraction 09 revealed 191 cluster or individual human proteins. We discarded albumin, hemoglobin chain proteins and immunoglobulins as these proteins were considered to be contaminants or not relevant. We compared our data with the dataset of Wijten et al. [25], who specifically reported APR proteoma and reduced the list to 52 individual proteins. Based on our results using HUVEC, we considered that the APR soluble factor was conserved among human and mouse. In silico, we aligned the obtained 52 human proteins and their mouse homologs, looking for proteins with the highest similarity (Table S1). Applying a stringent criterion of a minimum of 80% identical amino acid sequence between human and mouse homologs, we reduced our dataset to 18 candidate proteins (Table 1).

### 3.4. Von Willebrand Factor Induces Interleukin-6 Secretion by Liver Sinusoidal Endothelial Cells, But Its Absence in Activated Platelet Releasate Has No Impact on Interleukin-6 Secretion

**Figure 4:**
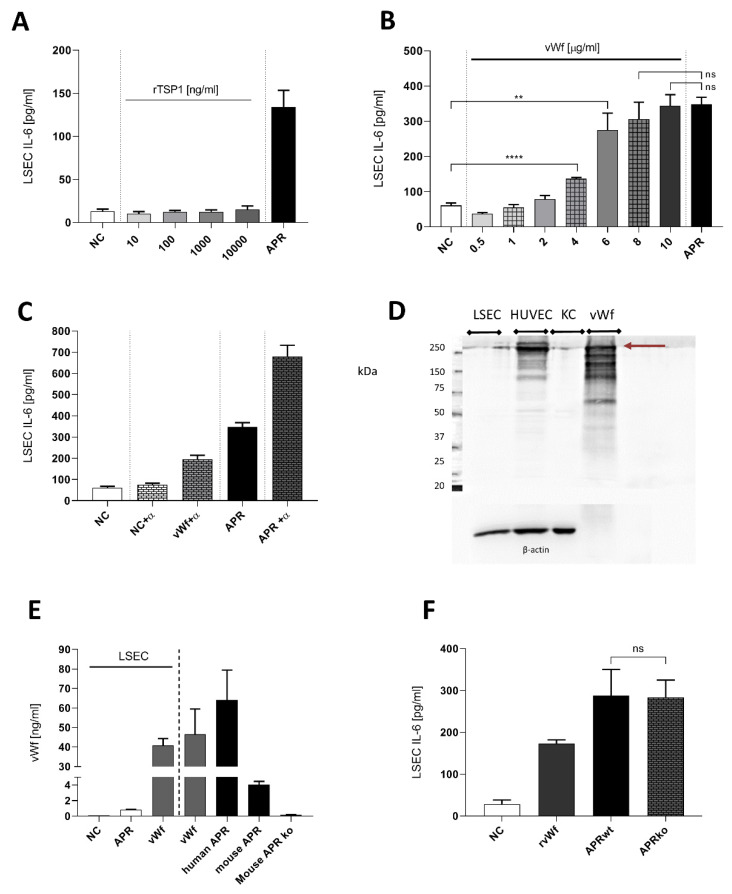
Characterization of soluble factors potentially inducing interleukin-6 secretion by liver sinusoidal endothelial cells. (**A**): Concentrations of thrombospondin 1 (TSP1) from 10 to 1000 ng/mL did not stimulate IL-6 secretion by LSEC. NC: negative control, tyrode buffer. (**B**): purified full-length human von Willebrand factor (vWF) was titrated on mouse LSEC. High concentrations of vWF resulted in an effect similar to human APR (positive control). NC: negative control, culture medium. (**C**): Neutralization of human APR or 8 μg/mL purified human vWF with anti-human vWF polyclonal antibody (α) 1/150. Combination of the antibody with APR or vWF resulted in an unexpected increase of IL-6 secretion by LSEC. NC: negative control, culture medium. (**D**): Western blot for expression of vWF in LSEC, HUVEC, KC as a positive control, human purified full-length vWF (2.5 μg). Contrarily, to HUVEC, LSEC and KC demonstrated low expression of vWF (250 kDa band, burgundy arrow). Purified full-length vWF exhibited several unspecific bands that were similar to HUVEC. Loading was controlled with β-actin. 7.5% acrylamide gel. *n* = 1 (**E**): Presence of mouse vWF was evaluated by ELISA in LSEC medium after treatment with APR. Human and mouse APR shows high amounts of vWF. NC: negative control, culture medium. (**F**): APR prepared from mouse double ko for vWF gene. APR was still able to induce IL-6 secretion by LSEC, 3 replicas. NC: negative control, culture medium. *n* = 1. **** *p* < 0.0001, ** *p*-value between 0.001, 0.01 (*t*-test),2 ns: non-significant. If not otherwise specified, presented data are representative of 2 or 3 independent experiments.

We first evaluated proteins known to be implicated in liver regeneration [26,27,28], including recombinant human TSP1 [29,30]. As shown in Figure 4A, TSP1 did not induce secretion of IL-6 by LSEC. As the role of vWF in liver regeneration had been described recently [27,28], we stimulated cultured LSEC with increasing concentrations of highly purified full-length human vWF (Figure 4B). We observed a statistically significant increase of IL-6 secretion by LSEC compared to control (Figure 4B). Moreover, when using concentrations at 8 or 10 µg/mL, IL-6 secretion level was similar to the stimulation with APR (Figure 4B). However, using a neutralization antibody to block soluble vWF in APR resulted in an unexpected increase of IL-6 production by LSEC (Figure 4C). To explore whether LSEC express vWF like other endothelial cells, which could contribute to IL-6 secretion induction by an autocrine loop, we assessed the presence of vWF in LSEC and its concentration after 24 h of culture. We observed that, in contrast to HUVEC, vWF was not expressed by LSEC (Figure 4D) and its concentration in LSEC supernatant remained very low, even after stimulation of LSEC with APR (Figure 4E). Finally, we used APR prepared from mice lacking vWF (vWF ko), which induced similar levels of IL-6 secretion by LSEC when compared to APR from wild-type mice (Figure 4F). This result indicated that vWF was not essential for the IL-6 production by LSEC.

### 3.5. Transforming Growth Factor β1 Is One of the Principal Activators in Platelet Releasate

**Figure 5:**
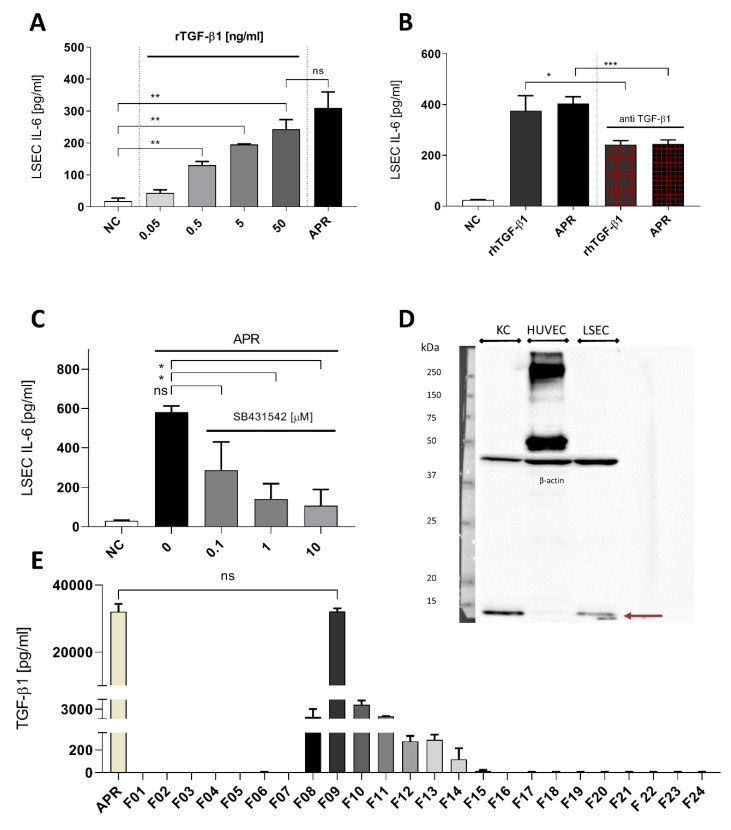
Transforming growth factor β1 is a strong candidate to activated platelet releasate soluble factor. (**A**): LSEC titration with recombinant human TGF-β1 induced a dose-response on IL-6 secretion, and at 50 ng/mL, there was no statistically significant difference with human APR. NC: negative control, culture medium. (**B**): Using anti-human TGF-β1, we were able to reduce the effect of APR or recombinant human TGF-β1 by 50%. Antibody concentration: 5 μg/mL, rTGF-β1 concentration: 5 ng/mL. NC: negative control, tyrode buffer. (**C**): TGF-β1 signaling blockade using the serine–threonine inhibitor SB431542 at increasing concentration. NC: negative control, DMSO (using DMSO with APR did not result in a decrease of IL-6 production). (**D**): Western blot for expression of TGF-β1 in KC, HUVEC and LSEC. TGF-β1 appeared in reduced condition as a monomer of 12.8 kDa (burgundy arrow) and was detected in KC and LSEC but not in HUVEC. Loading was controlled with β-actin. 10% acrylamide gel. *n* = 1. (**E**): TGF-β1 concentration in each fraction, obtained by ELISA, directly correlated with the IL-6 response pattern by LSEC. Indeed, fraction 09 showed a dramatic amount of TGF-β1 that were similar to the amounts found in non-fractionated human APR, *n* = 2. If not otherwise specified, presented data are representative of 2 or 3 independent experiments. *** *p*-value between 0.0001 and 0.001, ** *p*-value between 0.001 and 0.01, * *p*-value between 0.01 and 0.05 (*t*-test), ns: non-significant.

We then tested free recombinant human TGF-β1 for its ability to induce IL-6 secretion by LSEC (Figure 5A). Low concentration of TGF-β1 0.5 ng/mL as well as maximal concentration (50 ng/mL) induced IL-6 secretion respectively at 130.7 ± 11.7 and at 242.7 ± 30.5 pg/mL, representing a 13-fold increase over the baseline IL-6 release by LSEC (18.2 ± 8.9). There was no statistically significant difference between the effect of 50 ng/mL TGF-β1 and APR. To analyze the relevance of TGF-β1 in APR-induced IL-6 secretion, we used an anti-human TGF-β1. Thereby, we neutralized the APR-induced IL-6 secretion by 50% (Figure 5B). Moreover, the anti-human TGF-β1 antibody was not able to block completely the effect of human recombinant TGF-β1 (Figure 5B). To inhibit the signaling pathways of the TGF-β family, we used the serine–threonine inhibitor SB431542 of the TGF-β type II receptors (TβRII). LSEC treatment with this inhibitor at 1 μM resulted in a major decrease of APR effect on IL-6 secretion (Figure 5C). Using 10 μM of SB431542 reduced IL-6 secretion from 559.1 ± 92 pg/mL to 49.5 ± 10.6 pg/mL, demonstrating that the TGF-β1 signaling pathway was implicated in IL-6 secretion. As previous studies have described TGF-β1 secretion by LSEC [31], we performed Western blotting using LSEC and KC protein extractions to investigate whether TGF-β1 secreted by LSEC themselves is involved in IL-6 secretion. As shown in Figure 5D, we observed that, contrarily to KC, LSEC produced low levels of TGF-β1, which was also undetectable in HUVEC. Moreover, concentrations of TGF-β1 in LSEC supernatant after 24 h culture were low (data not shown). Importantly, preparations of human and mouse APR as well as fractions 09 obtained after gel filtration by FPLC (Figure 5E) showed high concentrations of TGF-β1, supporting our results where platelet-derived TGF-β1 induces IL-6 secretion by LSEC.

## 4. Discussion

Several studies in humans and rodents demonstrated a correlation between platelet counts and the capacity of the liver to regenerate [32,33]. The mechanism by which platelets stimulate liver regeneration remains largely unknown. Several previous studies revealed that IL-6 secretion occurs from endothelial cells after stimulation by lipopolysaccharide, protease-activated receptor-1 or-2 agonist, vascular endothelial growth factor (VEGF), IL-1 or tumor necrosis factor [34,35,36,37,38,39,40,41,42]. We have first tested several recombinant candidate proteins in our IL-6 secretion assay without observing an effect on IL6 production by LSEC [13] (see Table S2 summarizing all the assessed protein candidates). Using confocal microscopy, we detected Il-6 staining in LSEC following APR stimulation, suggesting that APR induced a de novo synthesis of IL-6. Moreover, we reported previously that APR stimulation of LSEC increased IL-6 mRNA expression [13]. Thereby, it is likely that APR increases IL-6 gene expression and de novo Il-6 synthesis in LSEC.

At early steps during the identification of potential active factors, we found that the factor was thermolabile, of high molecular weight and resistant to freezing, leading us to focus on proteins and to exclude smaller molecules e.g., serotonin and S1P. We also considered the possibility that soluble factors delivered by extracellular vesicles (EV) might be involved. Previous studies have shown that EV are highly implicated in liver physiological and pathological processes [3,43]. PMP have been reported to regulate gene expression in endothelial cells and to stimulate their production of IL-6 [44,45]. However, we did not observe an implication of platelet-derived EV in IL-6 secretion by LSEC.

Platelets are specific to mammals and present an evolutionary advantage for survival after injury, which probably explains their conserved functional structure [46]. We performed FPLC in order to fractionate human APR samples, obtained through PRP extraction using Regen BCT tubes. Using this technique, we reduced platelet manipulation, avoided their spontaneous activation and increased platelet yield compared to manual preparation. Silver staining of proteins in fractions obtained by FPLC loaded on polyacrylamide gels revealed that plasma proteins, such as albumin (65 kDa), were frequent contaminants. However, this had minimal impact on the outcome. Fractions that induced a statistically significant secretion of IL-6 by LSEC were analyzed by MS.

Although the human platelet proteome is constituted of up to 2800 proteins [47], human platelet granules contain 827 proteins, including granule-associated structural proteins [48]. However, it has been proposed that only 124 proteins are specifically released after human platelet activation constituting the APR [25]. We then selected candidates out of the identified proteins which matched with the 124 APR proteins reported by Wijten et al. (Table 1) [25]. The authors had analyzed released proteins, excluding granule-associated proteins. We used a stringent criteria of similarity to predict function and evolutionary origin of two proteins that are already considered as homologs in two close species and to increase the chance that proteins will cross-react between species. Therefore, we selected human proteins with at least 80% of identity based on protein amino acid sequence identity with their known mouse homologs and obtained 18 protein candidates.

The list included vWF, a factor released by activated endothelial cells and platelets [49]. During early liver regeneration, platelet accumulation and their activation in liver sinusoids are dependent on the presence of vWF [27,28]. In our assay, purified vWF induced the secretion of IL-6 by LSEC. Moreover, the release of IL-6 induced by APR incubated with an antibody neutralizing the vWF was unexpectedly increased. This might be explained by an activator effect of the antibody inducing conformational changes and unfolding of hidden binding sites [50,51]. The results suggest that antibody binding to vWF induced conformational changes, allowing binding to LSEC and leading to increased IL-6 secretion by LSEC. However, APR without vWF (prepared from vWF KO mice) actively induced IL-6 secretion, demonstrating that vWF in APR was not relevant in stimulating IL-6 release by LSEC. It remains open whether platelet-released vWF is relevant for IL-6 release in vivo since a conformational change could be induced through local protein association such as FVIII, released by LSEC [51].

Recombinant human TGF-β1 was as efficient as APR in inducing IL-6 secretion by LSEC. In the FPLC fraction 09, we observed a band under 20 kDa on polyacrylamide gel, suggesting the presence of TGF-β1 monomers of 12.5 kDa [52]. This signal was not present in other fractions. Indeed, TGF-β1 is released by platelets in a complexed form of 220 to 235 kDa (mature or latent form) with two other proteins (latency-associated peptide and latent TGF-beta binding protein) important for hindering or facilitating TGF-β1 activation [53]. TGF-β1 was absent from the MS analyzed fractions 08, 10 and 11, but latent TGF-beta binding protein was identified in both fractions 08 and 09. Measured free TGF-β1 in fraction 09 was above 30,000 pg/mL and coherent with the elicited maximal IL-6 response by LSEC that requires 50,000 pg/L recombinant TGF-β1.

To block the effect of TGF-β, we used SB431542, an inhibitor of the serine–threonine kinase of type I TGF-β receptor (TβRI). TβRI is phosphorylated by TβRII after ligand liaison with TβRI/TβRII (or ALK) and can further phosphorylate small mothers against decapentaplegic (SMAD) proteins that will assemble and recruit a transcriptional factor complex [54]. SB431542 induced a dramatic reduction of APR effect on IL-6 secretion by LSEC. Although SB431542 is a widely used inhibitor of TGFβ-1 signaling and is specific to ALK5 (the receptor of TGF-β), it also has an action on ALK4 and ALK7, the receptors of activin [55]. However, activin was not detected in the MS analyzed fractions [56].

TGF-β1 is implicated in numerous processes, including tissue healing [57]. In the liver, KC and LSEC produce TGF-β during homeostasis, while it is produced by HSC during inflammation and fibrosis [58,59]. TGF-β1 is a known activator of HSC, a terminator of hepatocyte proliferation, a suppressor of cytokine production by immune cells and also a direct inhibitor of macrophage maturation [54,60,61]. However, the effects of TGF-β have been described to be very different, even opposite, depending on cell types and conditions [62]. An implication of TGF-β1 in early liver regeneration may explain discrepancies reported on the role of this growth factor during the liver regeneration initiation phase [63,64]. Thus, TGF-β1-induced IL-6 production by LSEC might have the role of counteracting the effect of TGF-β1 itself. Indeed, as mentioned in the introduction, IL-6 promotes hepatocyte proliferation by a direct effect on hepatocytes [17] but also indirectly through the production of HGF by non-parenchymal cells such as the HSC, which was confirmed in an earlier work [13]. Moreover, TGF-β1 might turn KC toward a polarization state (M2c), where cells have anti-inflammatory proprieties and do not produce IL-6 [65]. In this state, KC IL-6 is not available anymore to stimulate HSC and hepatocytes. HSC are at the center of liver inflammation processes and can be seen as an additional factor connecting liver regeneration and inflammation.

**Figure 6:**
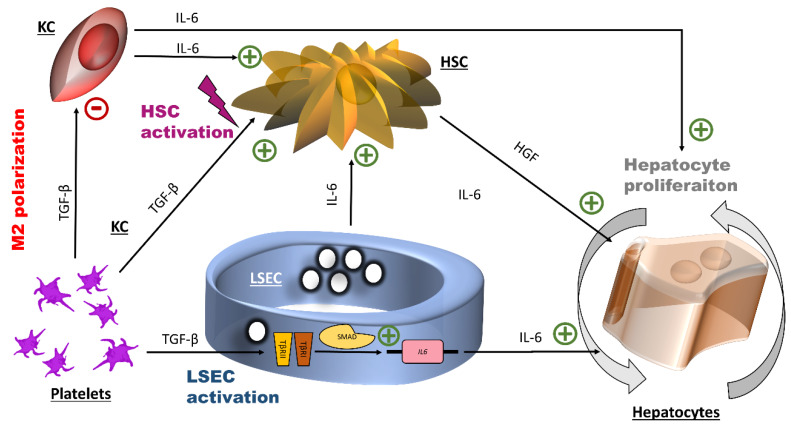
Potential effects of transforming growth factor β1 and interleukin-6 at the early stage of liver regeneration. The initial action of platelet TGF-β1 is the induction of the expression of the IL-6 gene by LSEC, probably through the canonical small mothers against decapentaplegic SMAD transcription factor complex. IL-6 promotes hepatocyte proliferation. Additionally, platelets TGF-β1 is connected to inflammation through its effects on hepatic stellate cells (HSC) (cell activation) and on Kuppfer cells (KC) (M2 polarization). HGF: hepatocyte growth factor, HSC: hepatic stellate cells, IL-6: interleukin-6, LSEC: liver sinusoidal endothelial cells, TGF- β1: transforming growth factor β1.

We identified TGF-β1 in APR to be responsible for inducing IL-6 secretion by LSEC via activation of the TGF-β signaling pathway. IL-6 is essential for priming hepatocytes by liver regeneration [17]. As summarized in Figure 6, our data suggest that TGF-β1 released through platelet activation during liver injury contributes to the onset of liver regeneration by increasing IL-6 levels by LSEC. However, the exact signaling pathway by which IL-6 is controlled following TGF-β receptor activation (canonical versus non-canonical) requires further confirmation. How TGF-β controls the IL-6 secretion by LSEC has not been extensively investigated. Park et al., using prostate cancer cells, showed that this control occurred through the canonical SMAD pathway [66]. Other TGF-β family members such as Activin A and bone morphogenic protein 6 also control IL-6 secretion through the SMAD pathway with the requirement of some co-signals [67,68]. Moreover, in endothelial cells, SMAD 4 regulates the production of angiopoietin-2 [69], a cardinal factor for autocrine stimulation of LSEC. This process could also be regulated by TGF-β in LSEC [31].

## 5. Conclusions

In this work, we demonstrated that TGF-β1 derived from mouse and human platelets specifically stimulate cultured mouse LSEC to release high amounts of IL-6. Further work will determine whether other soluble factors present in APR are implicated in this process. The physiological importance of IL-6 secretion by LSEC induced by platelet-derived TGF-β1 in early liver regeneration needs further in vivo investigation. However, this study suggests that the cytokine TGF-β1 plays an important role in initiating liver regeneration.

## Figures and Tables

**Table 1:** Human proteins presenting a high level of similarity with their mouse homologs. Retained human proteins for their high level of identity (minimum 80%) between mouse and human proteins after in silico amino acid sequence alignment. Listed proteins correspond to protein clusters or individual proteins. The peptide column indicates the number of peptides identified by MS analysis. MW: molecular weight; identity indicates the percentage of common amino acid sequences between human and mouse.

#	Gene Name	Protein	Peptide	MW (kDa)	Identity (%)
1	TGFB1	Transforming growth factor β-1	5	44	89.7
2	TGFBI	Transforming growth factor, β-induced	8	75	90.6
3	SERPINA3	Alpha-1-antichymotrypsin	22	48	na
4	SERPINC1	Antithrombin-III	4	53	87.3
5	C6	Complement factor 6	18	105	na
6	CFB	Complement factor B	22	86	83.6
7	FN1	Fibronectin	62	263	88.2
8	NID1	Nidogen-1	26	136	85
9	THBS1	Thrombospondin-1	50	129	94.8
10	VWF	Von Willebrand factor	29	309	83.2
11	CALU	Calumenin	3	37	98.1
12	CP	Ceruloplasmin	49	122	83.1
13	SPARC	Basement-membrane protein 40	4	35	92.4
14	TTR	Transthyretin	7	16	81.6
15	APLP2	Amyloid-like protein 2	8	87	85.5
16	FSTL1	Follistatin-related protein 1	2	35	91.9
17	MAN2A1	Alpha-mannosidase 2	2	131	80.4
18	SERPINA4	Kallistatin	2	49	na

## Supplementary Materials

The following are available online at https://www.mdpi.com/2073-4409/9/5/1311/s1, Figure S1: Human and mouse activated platelet releasates were both able to induce the production of interleukin-6 by liver sinusoidal endothelial cells or human umbilical vein endothelial cells; Figure S2: Platelet microparticles are not implicated in interleukin-6 secretion by liver sinusoidal endothelial cells; Table S1: Proteins identified in fraction 09; Table S2: Summary of proteins tested.

## Abbreviations

EV	Extracellular vesicles
FBS	Fetal bovine serum
HGF	Hepatocyte growth factor
HSC	Hepatic stellate cells
HUVEC	Human umbilical vein endothelial cells
IL-6	Interleukin-6
KC	Kupffer cells
LSEC	Liver sinusoidal endothelial cells
vWF	von Willebrand factor
PDGF	Platelet-derived growth factor
PH	Partial hepatectomy
PRP	Platelet rich plasma
SMAD	Small mothers against decapentaplegic
TGF-β	Transforming growth factor β
TβRI	Type I TGF-β receptor
TβRII	Type II TGF-β receptor
TSP1	Thrombospondin 1
VEGF	Vascular endothelial growth factor
