# Supplementary material for: Platelet Transforming Growth Factor-β1 Induces Liver Sinusoidal Endothelial Cells to Secrete Interleukin-6
Source: Cells. 2020 May 25;9(5):1311. doi: 10.3390/cells9051311 (PMC7290849; doi:10.3390/cells9051311)
Supplement: Supplementary file 1 [file cells-09-01311-s001.pdf]

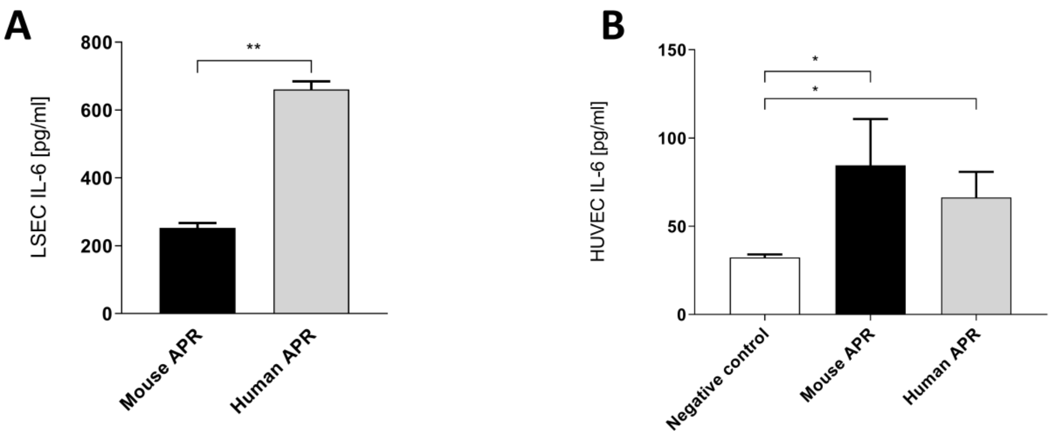

Figure S1. Human and mouse activated platelet releasates were both able to induce the production of interleukin-6 by liver sinusoidal endothelial cells or human umbilical vein endothelial cells. A: Human APR showed a stronger effect on LSEC IL-6 production than mouse APR. B: Cultured HUVEC incubated with mouse or human APR. NC: negative control, culture medium. Presented data are representative of 2 independent experiments. ** p value between 0.001 and 0.01. * p value between 0.01 and 0.05 (t-test).

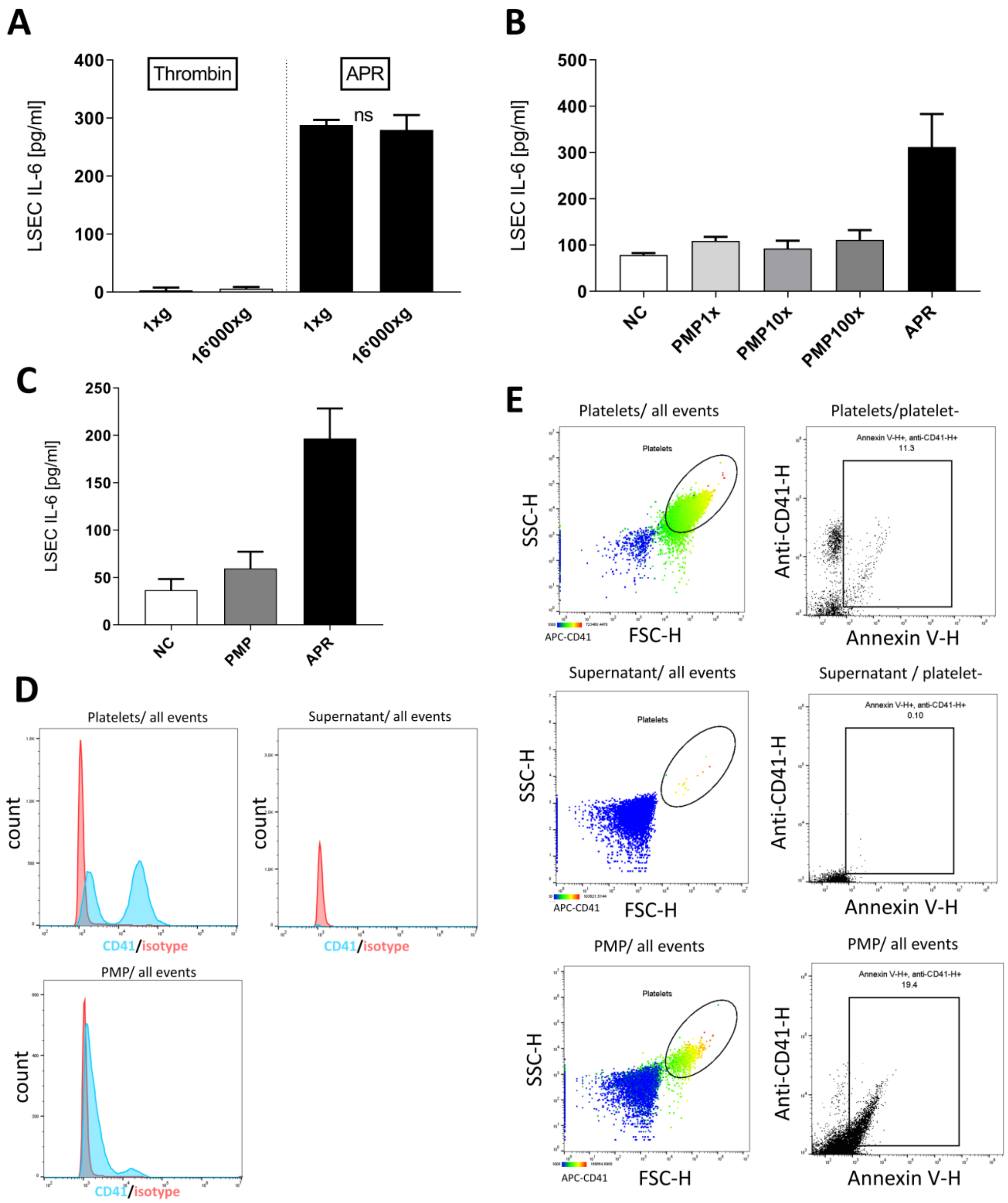

Figure S2. Platelet microparticles are not implicated in interleukin-6 secretion by liver sinusoidal endothelial cells. **A:** Mouse APR depleted of PMP induced the secretion of IL-6 by LSEC. NC: negative control, PBS, ns: not significant (t-test) **B:** TRAP-6 generated PMP at various concentration did not affect the release of IL-6 by LSEC, $n = 2$. NC: negative control, PBS. **C:** Platelet freeze-thawing-derived PMP did not induce the secretion of IL-6 by LSEC. NC: negative control, PBS. Ran in 4 to 5 replicas. **D:** Histogram of Isotype control and anti-human CD41 staining of human platelets, human PMP and their supernatant. E: platelets were identified by their side scatter and forward scatter morphology and high expression of CD41. Right: after inverted platelet gate (platelet-) selection, expression of CD41 was plotted against phosphatidylserine (annexin V) expression. PMP were identified as double positive for CD41 and annexin V. If not otherwise specified, presented data are representative of 2 to 4 independent experiments.

Table S1. Proteins identified in fraction 09. Dataset matching of mass spectroscopy (MS) dataset with human APR proteome, according to Wijten et al. (24). Listed proteins correspond to protein clusters or individual proteins. Peptide column indicates the number of peptides identified by MS analysis. MW: molecular weight, identity indicates the percentage of common amino acid sequences between human and mouse.

#	Gene name	Protein	Peptide	MW [kDa]	Identity [%]
1	ECM1	Extracellular matrix protein 1	10	61	66.8
2	TGFB1	Transforming growth factor beta-1	5	44	89.7
3	TGFBI	Transforming growth factor, beta-induced	8	75	90.6
4	CLEC3B	Tetranectin	2	23	79.2
5	ITIH3, ITH4	Inter-alpha-trypsin inhibitor	22,34	100, 103	84.9, 64.3
6	PLG	Plasminogen	4	91	79.1
7	SERPINA1	Alpha-1-antitrypsin	29	47	57.52
8	SERPINA3	Alpha-1-antichymotrypsin	22	48	na
9	SERPINC1	Antithrombin-III	4	53	87.3
10	SRGN	Serglycin	4	18	48.1
11	C3	Complement factor 3	152	187	76.8
12	C4A	Complement factor 4A	79	193	67.8
13	C6	Complement factor 6	18	105	na
14	C8B	Complement factor 8B	17	68	75.0
15	C9	Complement factor 9	4	63	57.6
16	CFB	Complement factor B	22	86	83.6
17	CFH	Complement factor H	33	139	61.2
18	PPBP	Platelet basic protein	2	14	47.7%

19	FGA, FGB,FGG	Fibrinogen	3, 4, 3	95, 56, 52	66,1, 81.4, 79.5
20	FN1	Fibronectin	62	263	88.2
21	HRG	Histidine-rich glycoprotein	12	60	60.9
22	LGALS3BP	Galectin-3-binding protein	7	65	67.7
23	NID1	Nidogen-1	26	136	85
24	NID2	Nidogen-2	13	151	78.5
25	THBS1	Thrombospondin-1			94.8
26	TNXB	Tenascin-X	5	458	71.9
27	VTN	Vitronectin	8	54	73.6
28	VWF	Von Willebrand factor	29	309	83.2
29	AHSG	Alpha-2-HS-glycoprotein	3	39	57.2
30	APOA1	Apolipoprotein A-I	53	31	65.2
31	CALU	Calumenin	3	37	98.1
32	CP	Ceruloplasmin	49	122	83.1
33	GC	Vitamin D-binding protein	14	53	77.7
34	HB	Haptoglobin	25	45	68.2
35	HPX	Hemopexin	18	52	73.4
36	SPARC	Basement-membrane protein 40	4	35	92.4
37	TF	Serotransferrin	31	77	72.5
38	TTR	Transthyretin	7	16	81.6
39	A1BG	Alpha-1B-glycoprotein	13	54	44.2
40	APCS	Serum amyloid P-component	9	25	68.8
41	APLP2	Amyloid-like protein 2	8	87	85.5
42	CLU	Clusterin	15	52	76.4

43	FSTL1	Follistatin-related protein 1	2	35	91.9
44	MAN2A1	Alpha-mannosidase 2	2	131	80.4
45	ORM2	Alpha-1-acid glycoprotein 2	5	24	42.5
46	PSAP	Prosaposin	4	58	63.4
47	QSOX1	Sulfhydryl oxidase 1	8	83	72.2
48	SEPP1	Selenoprotein P	2	43	70.2
49	PROS1	protein S	12	75	79.6
50	SERPINA4	Kallistatin	2	49	na
51	F5	Coagulation factor V	13	252	69.73
52	F13B	Coagulation factor XIIIB	4	76	76.4

Table S2. Summary of proteins tested. ANG-1: angiopoietin-1, ECM-1: extracellular matrix protein 1, PDGF-BB : platelet derived growth factor BB, SDF-1: stromal derived factor 1, TSP1: thrombospondin 1, TGF-β1: transforming growth factor β 1, vWf: Von Willebrand factor.

Protein	Tested concentrations	IL-6 modulation
CD154	Antibody blockade of APR	No
VEGF	5-100 [ng/mL]	No
SDF-1	25-100 [ng/mL]	No
ECM-1	2-200 [ng/mL]	No
PDGF-BB	1 to 100 [ng/mL]	No
ANG-1	1 to 1000 [ng/mL]	No
TSP1	0.1 to 1000 [ng/mL]	No
vWf	1 to 10 [μg/mL]	Yes
TGF-β1	0.01 to 500 [ng/mL]	Yes
